# Nd:YAG laser hyaloidotomy in the management of Premacular Subhyaloid Hemorrhage

**DOI:** 10.1186/s12886-016-0218-0

**Published:** 2016-04-18

**Authors:** Deepak Khadka, Sanjeeb Bhandari, Sanyam Bajimaya, Raba Thapa, Govinda Paudyal, Eli Pradhan

**Affiliations:** Tilganga Institute of Ophthalmology, Kathmandu, Nepal; Geta Eye Hospital, Dhangadi, Nepal

**Keywords:** Nd:YAG laser, Hyaloidotomy, Premacular hemorrhage, Subhyaloid hemorrhage

## Abstract

**Background:**

Premacular subhyaloid hemorrhage results in a sudden profound loss of vision. Among the modalities for its treatment, Nd:YAG laser hyaloidotomy is a non invasive method enabling rapid drainage of the obstructed macular area and improved vision within days. This study was aimed to evaluate the efficacy, visual outcome and complications following Nd:YAG laser hyaloidotomy for premacular subhyaloid hemorrhage.

**Methods:**

Patients with premacular subhyaloid hemorrhage of more than 3 disc diameters (DD) of various etiologies, attending Tilganga Institute of Ophthalmology, Nepal from August, 2014 to February, 2015, were included. A comprehensive ocular evaluation was conducted and fundus photographs were taken to measure the size of the subhyaloid hemorrhage. Optical coherence tomography (OCT) were performed before and after treatment and on subsequent follow up visits. Fundus fluorescence angiography was done whenever necessary. Q switched Nd:YAG laser was applied to create an opening in the posterior hyaloids membrane for draining subhyaloid hemorrhage. The main outcome measures were success rate in performing hyaloidotomy, drainage of subhyaloid blood into vitreous cavity and its resorption, improvement in visual acuity, need for further intervention and postoperative complications.

**Results:**

There were 21 eyes of 19 patients, 17(89.48 %) male and 2(10.52 %) female. In 3, premacular subhyaloid hemorrhage was bilateral. Mean age was 41.68 ± 17.08 years and a mean duration of symptoms 15.04 days. Mean pretreatment hemorrhage was 6.27DD. Nd:YAG laser hyaloidotomy was successful in 19 eyes(86.4 %). In 2 patients, one each with Eales’ disease and retinal vein occlusion the procedure was unsuccessful, necessitating pars plana vitrectomy, while in a case with proliferative diabetic retinopathy (PDR), vitrectomy was resorted for non clearing vitreous hemorrhage. Vision improved from a median of 3/60 pre-operatively to 6/6, at 6 months follow up. At 3 months, 2 patients with Eales’ disease, one developed tractional detachment at macula while the other, an epiretinal membrane. No other complications were noted at 6 months.

**Conclusion:**

Nd:YAG laser hyaloidotomy is an inexpensive, effective and a safe outpatient procedure for premacular subhyaloid hemorrhage, producing rapid drainage with restoration of visual function avoiding more invasive procedures and enabling early assessment of the underlying retina. The final visual prognosis however, rests on the underlying cause of the subhyaloid hemorrhage and any accompanying retinal changes.

## Background

Subhyaloid hemorrhage is a localized detachment of the vitreous from the retina due to the accumulation of blood [1]. When localized in the macular area, it results in sudden profound loss of vision. Subhyaloid premacular hemorrhage is typically characterized by a circumscribed, round or dumb-bell shaped, bright red mound of blood beneath the internal limiting membrane (ILM) or between the ILM and hyaloid face, in or near to the central macular area [2, 3]. It may occur in retinal vascular disorders, such as proliferative diabetic retinopathy [4], retinal vein occlusion [5], macroaneurysm [4], age-related macular degeneration [5] and arterio-venous communication of the retina; [6] hematological disorders as aplastic anemia and leukemia; [7] following laser in situ keratomileusis (LASIK); [8] or after retinal vascular rupture associated with physical exertion (valsalva) [2, 9–11], Terson’s syndrome [12] and Purtscher’s retinopathy [13]. Though spontaneous resolution occurs in most cases, this process takes several weeks or months depending on the thickness and total amount of blood present, often incapacitating to the patient when occurring bilaterally or in a one eyed patients. Moreover, it may result in permanent visual impairment due to pigmentary macular changes or formation of epiretinal membranes and toxic damage to the retina due to prolonged contact with hemoglobin and Iron [14].

Various techniques have been described to treat premacular subhyaloid hemorrhage. These include, observation, Nd:YAG laser hyaloidotomy [15], pneumatic displacement of hemorrhage by intravitreal injection of gas and tissue plasminogen activator [16] and pars plana vitrectomy [17]. Puncturing the posterior hyaloid face with Nd:YAG or green argon laser is a non invasive method, which enables the drainage of the extensive premacular subhyaloid hemorrhage into the vitreous, facilitates absorption of blood cells and improves vision within days by clearance of the obstructed macular area. In this study, we describe the etiologies, patient characteristics and outcome of Nd:YAG laser hyaloidotomy in 22 eyes of 19 patients presenting with premacular subhyaloid hemorrhage.

## Methods

This nonrandomized prospective interventional case series included patients with subhyaloid premacular hemorrhage of various etiologies attending Tilganga Institute of Ophthalmology, Nepal from August, 2014 to February, 2015. Only those patients with subhyaloid hemorrhage more than 3 disc diameters (DD) (by approximation and measured by the same person) and without significant vitreous opacities precluding the use of Nd:YAG laser, were included. Pretreatment and post-treatment assessment, conducted by trained retina specialists, included best corrected visual acuity (Snellen chart), slit lamp biomicroscopic examination with +90D (Volk) lens, intraocular pressure and fundus evaluation. Fundus photographs were taken to measure the size of the subhyaloid hemorrhage by comparing with disc dimensions. The horizontal and vertical diameters of the preretinal hemorrhage were measured in DD and averaged. Optical coherence tomography (OCT) were performed before and after treatment and on subsequent follow up visits. Fundus fluorescence angiography was performed whenever necessary.

After detailed discussion of management options, the procedure was explained to the patient and consent obtained. Mydriasis was achieved with tropicamide 0.5 % eye drop and topical anesthesia with lignocaine 4 % eye drops. Q switchedNd:YAG laser (Ellex Super Q, Ellex, USA) emitting single burst was delivered through a slit lamp delivery system using Goldman three mirror contact lens (Volk) to create an opening in the posterior hyaloid membrane near the inferior edge of the subhyaloid hemorrhage, avoiding retinal blood vessels and fovea but keeping a reasonably sufficient underlying cushion of blood to shield the underlying retina. Laser exposures were started with 5mj and then gradually increased 1mj each, until a perforation became visible at the surface and blood drainage under gravity into the vitreous cavity was evident. In those cases, where blood did not drain even after 8 shots, the procedure was abandoned. Patients were followed at 1 week, 6 weeks, 3 months and 6 months period.

The main outcome measures were success rate in performing hyaloidotomy, drainage of subhyaloid blood into vitreous cavity and its resorption, improvement in visual acuity, need for further intervention and postoperative complications. Institutional review board of Tilganga Institute of Ophthalmology approved this study.

## Results

**Fig. 1:**
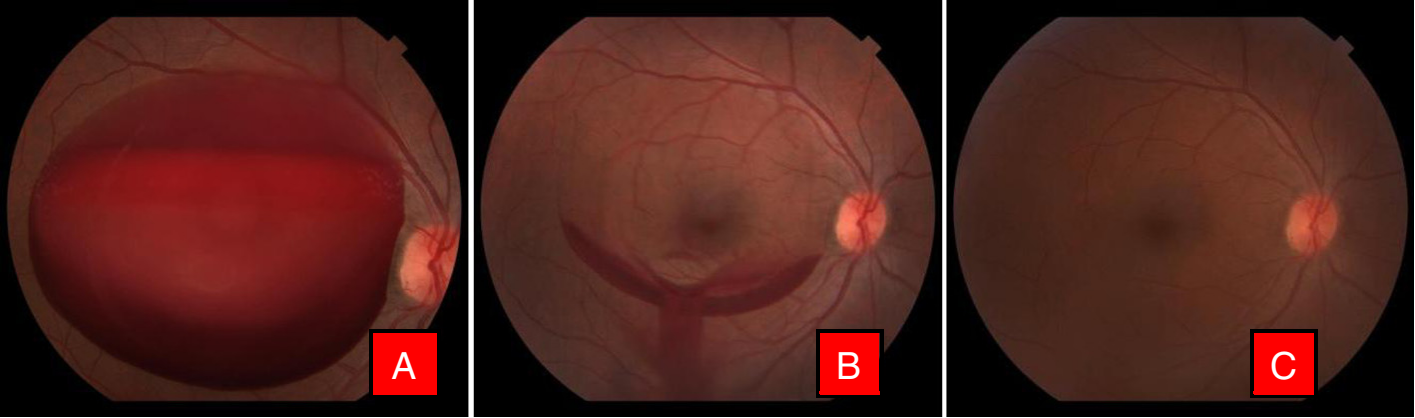
Color fundus photographs of a patient with Valsalva retinopathy. **a** Demonstrating boat shaped hemorrhage. **b** Same patient immediately after Nd: YAG laser hyaloidotomy. Note the draining premacular hemorrhage. **c** Fundus picture after 6 weeks

**Fig. 2:**
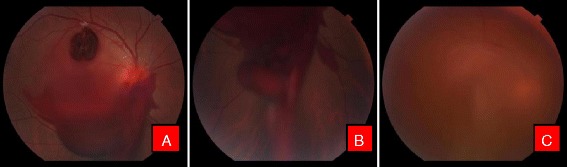
Color fundus photographs of a patient with ruptured macroaneurysm. **a** Before treatment. Note the macroaneurysm in superotemporal quadrant. **b** Immediately after Nd:YAG photodisruption with blood draining into vitreous cavity inferiorly (**c**). Resolving hemorrhage after 1 week

**Fig. 3:**
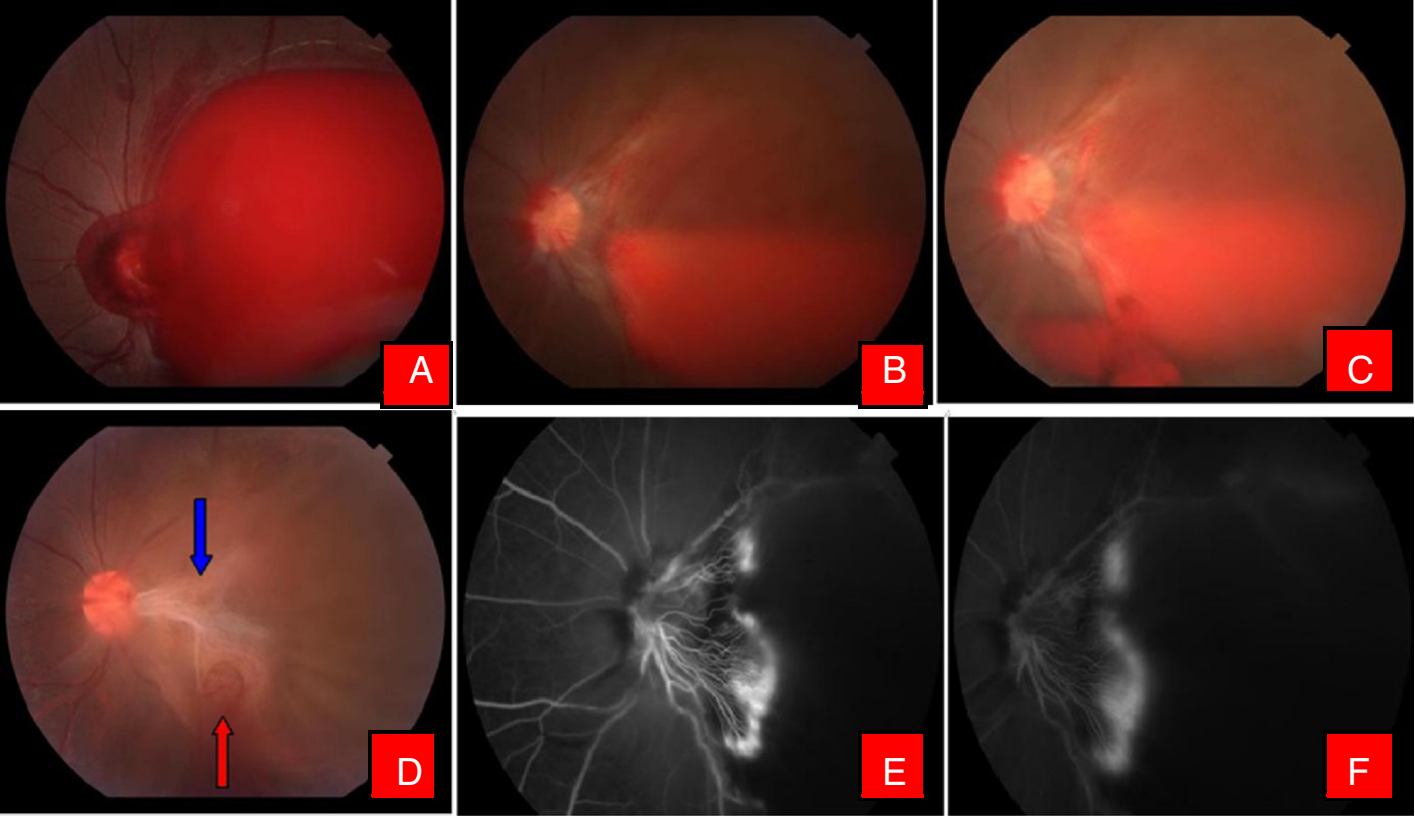
Color fundus photographs of a patient with Eales’ disease. **a** At the time of presentation. **b** Note the liquefied blood with neovascularisation of the disc. **c** Patient after Nd:YAG laser hyaloidotomy. Note the draining blood. **d** 6 weeks after hyaloidotomy. Note the tractional detachment at macula due to regressing neovascular frond (*Blue arrow*) and hyaloidotomy opening (*Red arrow*). **e** Fundus flurescein angiography (FFA) showing hyperflurescence due to neovascularisation of the disc. **f** Late phase FFA of the same patient with characteristic leakage of dye

**Fig. 4:**
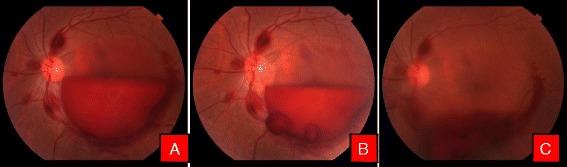
Color fundus photographs of a patient with leukemic retinopathy. **a** With multiple nerve fiber layer hemorrhage. **b** Immediately after Nd:YAG hyaloidotomy with two openings. **c** Day 1 after laser, with clearing premacular bleed

**Fig. 5:**
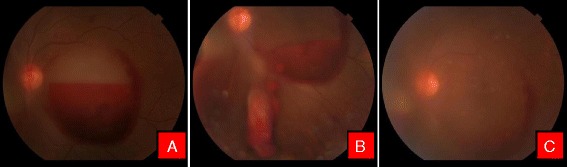
Color fundus photographs of a patient with high altitude retinopathy. **a** Characteristic boat shaped hemorrhage with multiple retinal hemorrhages. **b** Immediately after Nd:YAG laser with blood draining into vitreous cavity. **c** Clearing premacular hemorrhage at the end of 1 week

**Fig. 6:**
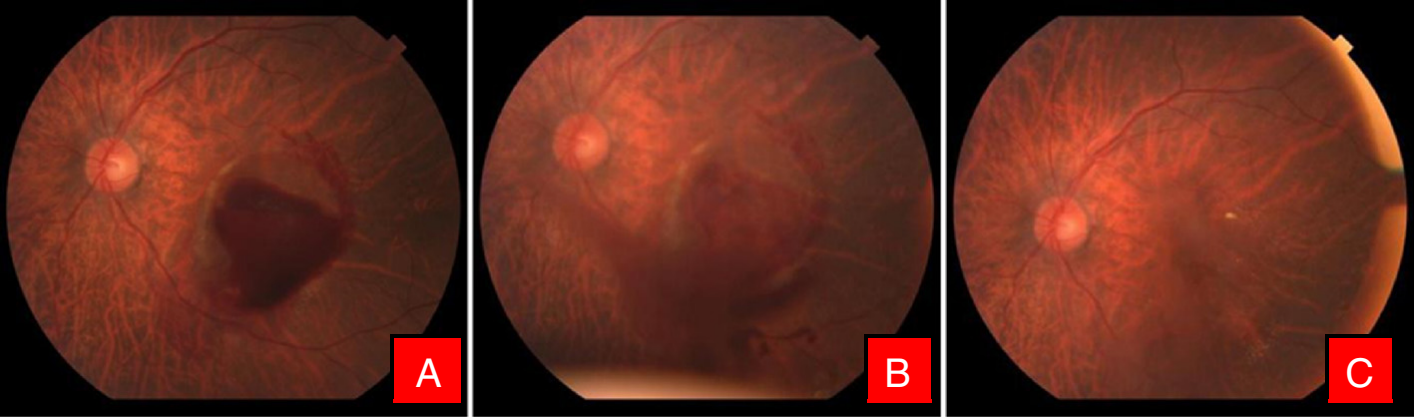
Color fundus photographs of a patient with CNVM. **a** Characteristic boat shaped hemorrhage. **b** Immediately after Nd Yag laser. **c** 3 months later after couple of intravitreal Bevacizumab injection

**Fig. 7:**
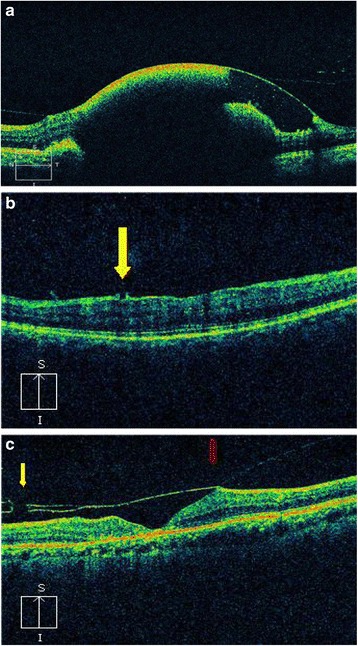
**a** Optical coherence tomogram (OCT) of a patient with Valsalva retinopathy showing hemorrhage under internal limiting membrane (ILM). **b** OCT of a patient with Valsalva retinopathy 6 weeks after laser photodisruption. Note the opening in ILM (*yellow arrow*). **c** OCT of a patient after Nd:YAG laser hyaloidotomy. Note the defect in posterior hyaloid membrane (yellow arrow) and the Vitreomacular traction (*red arrow*)

This study included twenty-two eyes of nineteen patients with premacular subhyaloid hemorrhage, 17 (89.48 %) were male and 2 (10.52 %) were female. In three patients premacular subhyaloid hemorrhage was bilateral. Mean age of the participants was 41.68 ± 17.08 years (range 18–76 years) with a mean duration of symptoms 15.04 days (range 1–49 days). Etiologies of premacular subhyaloid hemorrhage in 22 eyes were noted as valsalva retinopathy in 5 (22.72 %) [Fig. 1], ruptured retinal artery macroaneurysm in 2 (9.09 %) [Fig. 2], Eales’ disease in 5 (22.72 %) [Fig. 3a], Terson’s syndrome in 1 (4.54 %), proliferative diabetic retinopathy (PDR) in 4 (18.18 %), leukemic retinopathy in 2 (9.09 %) [Fig. 4], high altitude retinopathy in 1 (4.54 %) [Fig. 5], choroidal neovascular membrane (CNVM) in 1 (4.54 %) [Fig. 6] and branch retinal vein occlusion in 1 (4.54 %). The mean pretreatment hemorrhage as judged by fundus photography was 6.27 disc areas. Most of the cases of Valsalva retinopathy showed sub-ILM bleed [Fig. 7a] and one with true subhyaloid hemorrhage. One patient with Eales’ disease had both sub-ILM and subhyaloid component. Fundus fluorescence angiography was carried out in few patients for diagnostic purpose and for monitoring of therapeutic responses [Fig. 3e & f].

Preoperative visual acuity ranged from hand motion to 6/60. The mean number of laser shots were 2.5 (range 1–8) and the mean energy required to perform posterior hyaloidotomy was 6.54mj. After 1 week, visual acuity ranged from hand motion to 6/6 (Median 6/18), at 6 weeks follow up visual acuity ranged from hand motion to 6/6 (Median 6/9) and at 3 and 6 months follow up visual acuity ranged from 6/24 to 6/6 (Median 6/6). Nd-YAG laser hyaloidotomy with drainage of premacular subhyaloid hemorrhage was successful in 19 out of 22 eyes (86.4 %). In two patients, one with Eales’ disease (case 3) and the other with retinal vein occlusion (case 12), Nd:YAG laser hyaloidotomy was not able to drain subhyaloid hemorrhage necessitating pars plana vitrectomy while in a case with PDR (case 6), vitrectomy had to be performed for non clearing vitreous hemorrhage. During 6 weeks follow up, a patient with Eales’ disease (case 13), who had successful drainage of subhyaloid blood in the vitreous cavity, laser opening clinically evident on fundus evaluation, later developed tractional detachment at macula due to regressing neovascularisation frond, requiring pars plana vitrectomy [Fig. 3]. Additionally, an epiretinal membrane developed in a patient with Eales’ disease (case 17). No other complication including increase in intraocular pressure, retinal and choroidal hemorrhage, macular hole or retinal break formation was noted. Table 1 summarizes patients’ characteristics and post operative results.

**Table 1:** Nd: YAG Laser hyaloidotomy for premacular subhyaloid hemorrhage: Patient Characteristics

Case	Age(years)/Sex	Duration (Days)	Diagnosis	Eye	Size DD	Visual acuity					No of laser shots	Energy (mj)	Additional intervention/complication
						Pre Op	1 week	6 weeks	3 months	6 months			
1	76/M	3	Macroaneurysm	LE	10	6/60	6/36	6/24	6/24	6/24	2	5,6	
2	38/M	3	Valsalva Retinopathy	LE	5	CFCF	6/36	6/6	6/6	6/6	1	5	
3	30/M	2	Eales’ Disease	RE	≥10	HM	HM	HM	6/60^a^	6/12^a^	8	5,6,7,8,9,10,11,12	PPV
4	25/M	15	Terson’s Syndrome	RE	5	1/60	6/6	6/6	6/6	6/6	1	5	
5	42/M	10	Valsalva Retinopathy	LE	4	1/60	-	6/9	6/9	6/9	1	5	
6	49/M	49	PDR	LE	7	1/60	5/60	HM	6/60^a^	6/12^a^	6	5,6,7,8,9,10	PPV
7	44/M	14	PDR	RE	5	3/60	6/18	6/18	6/18	6/18	2	5,6	
		18	PDR	LE	7	4/60	6/9	6/9	6/9	6/9	1	5	
8	37/M	1	Valsalva Retinopathy	RE	8	HM	6/6	6/6	6/6	6/6	1	5	
9	21/M	10	Eales’ Disease	RE	4	6/60	6/6	6/6	6/6	6/6	1	5	
		10	Eales’ Disease	LE	5	6/60	6/9	6/9	6/9	6/9	3	5,6,7	
10	48/M	4	Valsalva Retinopathy	LE	10	HM	6/36	6/9	6/9	6/9	1	5	
11	68/F	15	Valsalva Retinopathy	RE	5	5/60	6/24	6/6	6/6	6/6	2	5,6	
12	69/M	30	Retinal vein Occlusion	LE	≥10	CFCF	HM	HM	6/9^a^	6/9^a^	8	5,6,7,8,9,10,11,12	PPV
13	18/M	2	Eales’ Disease	LE	8	HM	6/36	6/36	6/9^a^	6/9^a^	2	5,6	PPV
14	21/M	10	Leukemic Retinopathy	RE	4	6/60	6/6	6/6	6/6	6/6	1	5	
		12	Leukemic Retinopathy	LE	5	6/60	6/9	6/6	6/6	6/6	1	5	
15	28/M	6	High Altitude Retinopathy	RE	5	1/60	6/12	6/6	6/6	6/6	1	5	
16	54/M	15	Macroaneurysm	LE	8	1/60	6/18	6/18	6/18	6/18	1	5	
17	21/M	45	Eales’ Disease	RE	7	HM	6/24	6/18	6/12	6/12	5	5,6,7,8,9	ERM
18	48/F	45	PDR	LE	8	6/60	6/18	6/18	6/18	6/18	4	5,6,7,8	
19	55/M	12	CNVM	LE	4	CFCF	6/36	6/36	6/12	6/12	2	5,6	

*M* Male, *F* Female, *RE* Right Eye, *LE* Left Eye, *DD* Disc diameter, *HM* Hand Motion, *CFCF* Counting finger close to face, *ERM* Epiretinal membrane, *PPV* Pars plana vitrectomy: ^a^- Not analysed for outcomes of hyaloidotomy

Interestingly, apart from a case of high altitude retinopathy (case 15) included in the series, we observed 2 more cases of premacular subhyaloid hemorrhage ( <3 DD) with high altitude exposure. All the three patients had returned from expedition to the Everest Base Camp in Nepal (at an altitude of 5364 metres).

As the size of the hemorrhage was less than 3 DD in the latter 2 patients, after explanation of the management options, they opted for a conservative approach. Unfortunately, they were visitors to Nepal and they returned to their respective countries, with no available further data. Table 2 describes characteristics of patients with high altitude exposure.

**Table 2:** Characteristics of patients with high altitude exposure

Serial no	Age(years)/Sex	Duration (Days)	Diagnosis	Eye	Size DD	Visual acuity	Remarks
1	28/M	6	High Altitude Retinopathy	RE	5	1/60	Received Nd:YAG hyaloidotomy^a^
2	25/M	6	High Altitude Retinopathy	LE	<3	6/60	Opted for conservative management
3	27/F	6	High Altitude Retinopathy	RE	<3	5/60	Opted for conservative management

*M* Male, *RE* Right Eye, *LE* Left Eye, *DD* Disc diameter; ^a^Case no 15 in Table 1

## Discussion

Premacular subhyaloid hemorrhage produces sudden profound loss of vision that may be prolonged if untreated [1, 9]. Various therapeutic options are available including observation, pneumatic displacement, vitrectomy and Nd:YAG laser hyaloidotomy. Spontaneous resorption of blood entrapped in the subhyaloid space may take few weeks to several months. A slowly resolving subhyaloid hemorrhage might cause permanent visual loss due to pigmentary macular changes or growth of epiretinal membrane, and also prolongs the contact of the retina with blood, hemoglobin and iron, possibly causing toxic damage to the retina and reducing visual function, which may be irreversible [18, 19]. Early treatment allows rapid restoration of vision and visualization of the macular area with expedited access for fluorescein angiography and macular photocoagulation. Moreover, a quick recovery from this incapacitating vision deprivation is desired by most patients owing to their younger age. This becomes particularly important for one eyed patients, patient with poor vision in their fellow eye or in bilateral cases.

Nd:YAG laser assisted drainage of a premacular subhyaloid hemorrhage and subinternal limiting membrane (ILM) hemorrhage was described in the 1980’s [15, 20]. Since then, there have been various studies describing this procedure with varying degree of success. Timing is quite crucial, for successful drainage of blood through hyaloidotomy opening. Previous studies have shown that the premacular hemorrhage may clot [7, 8, 10, 21] or may re-bleed after successful drainage [7]. In this series, despite a successful opening in the posterior hyaloid, premacular hemorrhage in a patient with Eales’ disease and in the other with retinal vein occlusion, failed to drain into the vitreous, necessitating a pars plana vitrectomy. Notably, these patients had premacular hemorrhages greater than 10 discs diameter in size, suggesting that the size of the hemorrhage as a prognostic factor for the success of the procedure. In the studies those achieved successful drainage of premacular hemorrhage, visual acuity improved within days in most of the patients, the degree of improvement depended on the underlying and pre-existing macular damage [9, 15, 17, 18]. As in the previous studies [10, 11, 22, 23], valsalva retinopathy and Terson’s syndrome in this series showed the best results owing to the lack of underlying pathology. Additionally, in this study, a patient with high altitude retinopathy showed good improvement in visual acuity and we had also observed premacular subhyaloid hemorrhage in 2 more patients who had a history of ascension to high altitude. We were not able to find any study describing this as an etiology and the role of Nd:YAG laser hyaloidotomy in the management of this condition.

Two patients with ruptured macroaneurysm in this series did not gain complete visual recovery owing to the associated macular pathology, a finding consistent with the study of Ahmadabadi [12]. Patient with leukemic retinopathy and CNVM did well with Nd:YAG laser hyaloidotomy however, patient with CNVM required several doses of intravitreal Bevacizumab following successful Nd:YAG laser [7]. Premacular subhyaloid hemorrhage associated with PDR yielded mixed results, consistent with the study of Ahmadabadi [12]. Among the patients with subhyaloid hemorrhage with PDR, three achieved improvement in visual acuity, while in one Nd:YAG was not success resorting to pars plana vitrectomy. Interestingly in this study, two patients with symptoms of more than 45 days, Nd:YAG laser hyaloidotomy was successful to drain premacular blood into the vitreous cavity. This signifies that the state of blood in the premacular area is important rather than the duration of symptoms. In this study, we did not compare the success of Nd:YAG laser hyaloidotomy with the duration of symptoms as in other series [7, 8, 10, 12, 15, 22, 23]. In this series hyaloidotomy was successful in 19 eyes(86.4 %) with visual improvement in all successful cases, though a patient with Eales’ disease after drainage of premacular hemorrhage developed tractional retinal detachment at macula at 6 weeks follow up requiring a pars plana vitrectomy with recovery to 6/6.

Ulbig et al., in their case series of 21 patients, have reported a success rate of 76.2 % with good visualization of the macula within one month [10]. Complications up to 6 months after laser treatment in their case series, were negligible with a retinal detachment in a myopic patient with bilateral breaks and a single additional eye that developed a macular hole. Probably the breaks were secondary to myopic changes, which were not related to the laser while the macular hole that developed in the latter eye could be because the photodisruptive effect might have been too near to the macula. Epiretinal membrane formation (ERM) and contraction of ILM can occur following laser disruption of premacular hemorrhage. Kuruvilla et al. and Ahmadabadi et al. reported formation of epiretinal membrane after laser photodisruption [12, 22]. In this series, in patients with Eales’ disease, one developed ERM and another tractional macular detachment, 6 weeks after treatment of premacular hemorrhage with photodisruption. Kwok et al. on analysis of such membranes subsequently removed surgically had found hemosiderin deposits under the contracted ILM and fine glial ERM on the outer surface [2]. However, the authors of some series did not notice complications 6 months after Nd:YAG laser assisted hyaloidotomy for premacular subhyaloid hemorrhage [7, 11, 14, 23, 24].

Energy levels ranging from 2.5-50 mJ has been mentioned in the literature [3, 4, 7, 10–12, 15, 20, 22, 23, 25]. Gabel et al. reported use of 50 mJ energy without evidence of retinal burn [20]. Puthalath et al. [25] reported stretch burns to the hyaloid before YAG disruption however, this study did not find the step necessary for successful opening of posterior hyaloid. On analyzing the OCT, besides opening in the internal limiting membrane, no damage to the underlying retina was observed [Fig. 7], except in those cases with Eales’ disease where we believe the formation of the ERM and tractional macular detachment to be a part of the natural disease process due to regressing fibrovascular band.

Good clinical judgment, appropriate positioning of hyaloidotomy and use of the lowest possible energy level seems prudent for performing Nd:YAG laser photodisruption for premacular subhyaloid hemorrhage. It has been advocated that the size of the hemorrhage less than 3 disc diameter should not be subjected to photodisruptive laser for safety reasons. This size helps to increase the cushion effect of the hemorrhage in order to avoid inadvertent retinal damage by the photodisruptive laser [10]. There are few universal recommendations for posterior Nd:YAG laser hyaloidotomy. If possible, always drain from a region distant enough from the inferior border where there is significant hemorrhagic elevation. Furthermore, choosing a drainage area at a location away from the fovea and major blood vessels seems sensible. Depending on the location of the hemorrhage, and to avoid fovea, it is sometimes safer to drain blood from the lateral aspect of the hemorrhage and position the head so that the blood will drain into the vitreous cavity under the influence of the gravity [22]. It is best to start the treatment using the least amount of energy and then gradually increase the energy level until an adequate opening becomes visible. In this study, we experienced that it was better to start with 5 mJ energy and then gradually increase by 1 mJ each step to 12 mJ. If this energy level was not able to produce a puncture on the surface of the subhyaloid hemorrhage, it was less likely that it would perforate with increasing energy.

## Conclusion

As observed in our study, premacular subhyaloid hemorrhage can also occur with high altitude exposure, besides the other causes that have been described. In accordance with this and previous studies, Nd:YAG laser hyaloidotomy can be appreciated to be an inexpensive, effective and a safe outpatient procedure for the treatment of premacular subhyaloid hemorrhage. It produces rapid drainage of blood with restoration of visual function which would otherwise warrant more invasive vitreoretinal procedures and its associated serious complications. This is particularly beneficial for patients with poor vision in the fellow eye and patients requiring rapid visual rehabilitation to be able to continue their work. Besides, it also allows early assessment of the underlying retina with expedited access for macular photocoagulation and the avoidance of vitrectomy. The final visual prognosis however, rests on the underlying cause of the subhyaloid hemorrhage and any accompanying retinal changes. Long term surveillance of Nd:YAG laser treated cases and further randomized controlled clinical trials are required to compare the treatment with other modalities.

### Ethics approval and consent to participate

This study was approved by the Research Ethics Review Board of the Tilganga Institute of Ophthalmology. A written informed consent was obtained from all the participants after the study protocol had been explained.

### Consent to publish

A written informed consent to publish person’s data was sought along with the consent for participation into the study.

### Availability of data and materials

Data can be shared upon request.

## Abbreviations

CNVM: choroidal neovascular membrane
DD: disc diameters
ERM: epiretinal membrane
ILM: internal limiting membrane
LASIK: laser in situ keratomileusis
OCT: optical coherence tomography
PDR: proliferative diabetic retinopathy
